# Overexpression of TopBP1, a canonical ATR/Chk1 activator, paradoxically hinders ATR/Chk1 activation in cancer

**DOI:** 10.1016/j.jbc.2021.100382

**Published:** 2021-02-05

**Authors:** Kang Liu, Joshua D. Graves, Fang-Tsyr Lin, Weei-Chin Lin

**Affiliations:** 1 Section of Hematology/Oncology, Department of Medicine, Baylor College of Medicine, Houston, Texas, USA; 2 Department of Molecular and Cellular Biology, Baylor College of Medicine, Houston, Texas, USA

**Keywords:** TopBP1, ATR, Chk1, hormesis, AAD, ATR-activating domain, ATR, Ataxia telangiectasia mutated and Rad3 related, ATRIP, ATR-interacting protein, BRCT, BRCA1 carboxyl-terminal, CNA, copy number alteration, HU, hydroxyurea, MOI, multiplicity of infection, RPA, replication protein A, SCNA, somatic copy number alteration, TopBP1, topoisomerase IIβ-binding protein 1, TCGA, The Cancer Genome Atlas Program

## Abstract

Topoisomerase IIβ-binding protein 1 (TopBP1) is involved in cellular replication among other functions and is known to activate ATR/Chk1 during replicative stress. TopBP1 is also expressed at high levels in many cancers. However, the impact of TopBP1 overexpression on ATR/Chk1 activation and cancer development has not been investigated. Here we demonstrate that the degree of ATR/Chk1 activation is regulated by TopBP1 in a biphasic, concentration-dependent manner in a nontransformed MCF10A cell line and several cancer cell lines, including H1299, MDA-MB468, and U2OS. At low levels, TopBP1 activates ATR/Chk1, but once TopBP1 protein accumulates above an optimal level, it paradoxically leads to lower activation of ATR/Chk1. This is due to the perturbation of ATR–TopBP1 interaction and ATR chromatin loading by excessive TopBP1. Overexpression of TopBP1 thus hinders the ATR/Chk1 checkpoint response, leading to the impairment of genome integrity as demonstrated by the cytokinesis-block micronucleus assay. In contrast, moderate depletion of TopBP1 by shRNA in TopBP1-overexpressing cancer cells enhanced ATR/Chk1 activation and S-phase checkpoint response after replicative stress. The clinical significance of these findings is supported by an association between TopBP1 overexpression and genome instability in many types of human cancer. Taken together, our study illustrates an unexpected relationship between the levels of TopBP1 and the final functional outcome and suggests TopBP1 overexpression as a new mechanism directly contributing to genomic instability during tumorigenesis.

Maintenance of genome stability is an essential task in the propagation of life. The process of DNA replication during S phase poses the most vulnerable period in the cell cycle to both exogenous insults and endogenous replication errors. Thus, the S-phase checkpoint is of critical importance for the maintenance of genome stability. In mammalian cells, the S-phase checkpoint is mediated by the ATR/Chk1 pathway.

Ataxia telangiectasia mutated and Rad3 related (ATR) is a key sensor for damage or anomaly on replication forks, which phosphorylates Chk1 to halt DNA replication and cell cycle progression when cells encounter genomic insults. Activation of ATR depends on topoisomerase IIβ-binding protein 1 (TopBP1) (1) and Ewing's tumor-associated antigen 1 (2, 3, 4). TopBP1 contains nine BRCA1 carboxyl terminal (BRCT) domains. TopBP1 is involved in DNA replication, ATR checkpoint activation, DNA repair, mitosis, and transcriptional regulation (5). The ATR-activating function of TopBP1 is mediated by its ATR-activating domain (AAD) residing between its sixth and seventh BRCT domains (1). In response to replicative stress, replication protein A (RPA) binds to single-stranded DNA (ssDNA) and recruits ATR-interacting protein (ATRIP)/ATR. TopBP1 is also recruited to stalled replication forks *via* RPA-ssDNA (6) to interact with ATR–ATRIP. In addition, TopBP1 interacts with the Rad9-Hus1-Rad1 (9-1-1) clamp *via* its first and second BRCT domains (7). Ultimately, TopBP1 forms complex with both 9-1-1 and ATR–ATRIP on RPA-ssDNA to activate ATR through its AAD (8). Given the pivotal role of TopBP1 for ATR activation, ATR/Chk1 activation can be controlled through the regulation of TopBP1.

TopBP1 can be phosphorylated by Akt at Ser1159, which induces its oligomerization through TopBP1-BRCT7/8 domains (9). Oligomerization of TopBP1 inhibits its binding to ATR and therefore inhibits ATR activation (10). Oligomerization of TopBP1 also inhibits its binding to Treslin and prevents reinitiation of DNA replication in S/G2 phases (11). However, at the same time, oligomerization of TopBP1 induces its binding to E2F1 and MIZ1, thereby inhibiting E2F1-dependent apoptosis as well as MIZ1-dependent p21^Cip1^ expression (9). The ATR-activating function of TopBP1 can also be inhibited by mutant p53 (12). Many p53 mutants can bind TopBP1 and attenuate the checkpoint response by inducing TopBP1 oligomerization independent of Akt action.

TopBP1 is an E2F target, and its mRNA reaches peak levels during S phase of the cell cycle (13, 14). Many cancer cells express high levels of TopBP1 (15, 16, 17), in part due to deregulation of the Rb/E2F pathway. Here we investigated how TopBP1 overexpression may affect the ATR/Chk1 activation in cancer cells. It is surprising that we found that the amplitude of ATR/Chk1 activation is regulated by TopBP1 in a biphasic concentration-dependent manner, *i.e.*, at low levels, TopBP1 activates ATR/Chk1; but when TopBP1 levels reach a turning point, it paradoxically leads to a lower degree of ATR/Chk1 activation. As such, some cancer cells may not have appropriate checkpoint response owing to a high level of TopBP1. Indeed, TopBP1 overexpression is associated with genome instability in many types of cancer.

## Results

### The relationship between TopBP1 levels and the amplitude of ATR/Chk1 activation is a biphasic (hormesis) concentration-dependent response

**Figure 1:**
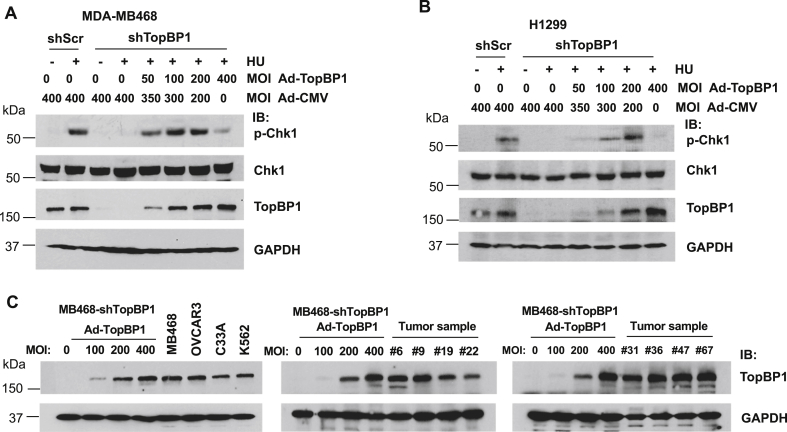
**High levels of TopBP1 in cancer cells paradoxically leads to attenuation of ATR/Chk1 response after hydroxyurea treatment**. *A*, MDA-MB468 cells stably expressing a scrambled shRNA (shScr) or a TopBP1 shRNA (shTopBP1) were infected with control Ad-CMV and/or Ad-TopBP1 adenoviruses at the indicated MOI, such that the total amount of virus was kept at 400 MOI for all samples. After 48 h, cells were treated with vehicle (dimethyl sulfoxide) or hydroxyurea (HU, 2 mM) for 5 h and then were harvested for Western blot analysis. *B*, H1299 cells stably expressing a scrambled shRNA (shScr) or a TopBP1 shRNA (shTopBP1) were infected with either Ad-CMV or Ad-TopBP1 at the indicated MOI. After 48 h, cells were treated with HU (2 mM for 5 h) as in (*A*). *C*, MDA-MB468-shTopBP1 stable cells were infected with increasing titers of Ad-TopBP1, and TopBP1 expressed in these cells were compared with the endogenous TopBP1 expressed in various cell lines (MDA-MB468, OVCAR3, C33A, and K562 cells) or primary breast cancer tissues. The numbers (#) are anonymous breast cancer patient ID numbers (15, 16). MOI, multiplicity of infection.

To determine how TopBP1 protein levels affect the intensity of Chk1 activation, we first knocked down TopBP1 in MDA-MB468, a triple-negative breast cancer cell line, and then reconstituted TopBP1 to different expression levels by infecting cells with a recombinant adenovirus Ad-TopBP1 at various multiplicity of infection (MOI). The total amounts of adenovirus were kept constant by adding a recombinant adenovirus Ad-CMV that harbors an empty vector. Cells were then treated with hydroxyurea (HU), a replication stress-inducing drug, and immunoblotting was performed to determine the levels of phospho-Chk1 (Ser345), which serves as a readout of Chk1 activity. Consistent with our prior report (12), the phosphorylation of Chk1 was almost completely blocked when TopBP1 was fully depleted but was gradually induced by adding TopBP1 in a concentration-dependent manner. It is surprising that, when cells were infected with Ad-TopBP1 at an MOI of 400, TopBP1 expression reached a level higher than that in control cells and the level of phosphorylated Chk1 dropped to a level significantly lower than that in the HU-treated shScr control cells (Fig. 1*A*). This phenomenon was also observed in another cancer cell line, H1299 (Fig. 1*B*). These data suggest that, although TopBP1 is essential for Chk1 phosphorylation in response to DNA damage, when TopBP1 is accumulated beyond an optimal level, it could instead have an inhibitory effect on Chk1 activation. This biphasic (hormesis or inverted U) dose response described in toxicology is characterized by a low-dose stimulation and high-dose inhibition to chemicals of interest, *i.e.*, existence of a sweet spot beyond which more chemicals paradoxically lead to a declining response. Many cancer cells express high levels of TopBP1 in part due to deregulation of the Rb/E2F pathway (15, 16, 17). If the levels of TopBP1 are higher than the optimal level for ATR activation, these cancer cells may, to some extent, become defective in checkpoint response to replicative stress.

To determine whether the levels of TopBP1 in those TopBP1-depleted cells infected with high titers of Ad-TopBP1 were still within physiological ranges compared with the endogenous TopBP1 protein levels in other cancer cells and tissues, we compared TopBP1 expression among these cells and three commonly used cancer cell lines, including OVCAR3, C33A, and K562 cells (Fig. 1*C*, *left panel*). We also compared their levels with the endogenous TopBP1 levels in eight primary breast cancer fresh-frozen samples that have been shown to express high levels of TopBP1 in a prior study (15) (Fig. 1*C*, *right two panels*). It appears that the levels of TopBP1 in the Ad-TopBP1-infected MDA-MB468-shTopBP1 cells were either comparable with or within twofold higher or lower than those expressed in the three cancer lines or tumor tissues. Thus, the levels of TopBP1 that can cause a paradoxically inhibitory effect on Chk1 activation are within the physiological range seen in many cancer cells.

### A modest reduction of TopBP1 expression in MDA-MB468 cells paradoxically potentiates ATR/Chk1 response, whereas highly efficient knockdown of TopBP1 blocks ATR/Chk1 activation

**Figure 2:**
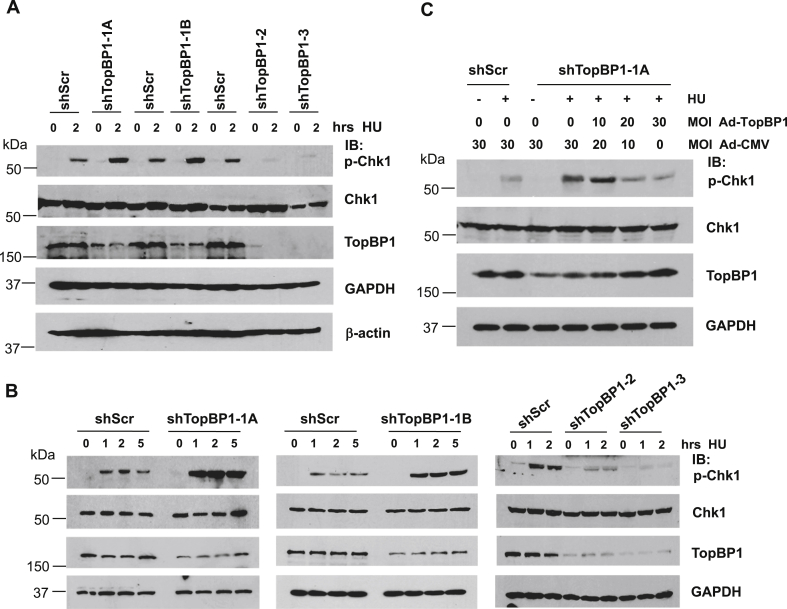
**A biphasic concentration-dependent effect of TopBP1 on hydroxyurea-induced Chk1 activation**. *A*, MDA-MB468 cells stably expressing a scrambled shRNA or a TopBP1 shRNA with modest (shTopBP1-1A and -1B) or high-efficient (shTopBP1-2 and -3) knockdown effect were treated with either DMSO or HU (2 mM) for 2 h. Cells were harvested and subjected to Western blot analysis as indicated. *B*, MDA-MB468 cells stably expressing a scrambled shRNA or a TopBP1 shRNA with modest or great depletion were treated with either DMSO or HU (2 mM) for various times and then harvested for immunoblotting. *C*, MDA-MB468 cells stably expressing a scrambled shRNA or a TopBP1 shRNA with modest depletion were infected with either Ad-CMV and/or Ad-TopBP1 at the indicated multiplicity of infection. After 48 h, cells were treated with either DMSO or HU (2 mM) for 5 h and then harvested for Western Blotting. DMSO, dimethyl sulfoxide; HU, hydroxyurea.

The data in Figure 1, *A* and *B* suggest that the effect of TopBP1 on p-Chk1 activation is biphasic concentration dependent, *i.e.*, an optimal concentration of TopBP1 is required for maximal ATR/Chk1 response. To further investigate this possibility, we stably knocked down TopBP1 to various degrees in MDA-MB468 cells that express TopBP1 at the highest level among a panel of breast cancer cell lines (18). We treated these stable cells with HU for 2 h and then compared the Chk1 phosphorylation by immunoblotting. Indeed, knockdown of TopBP1 to a mild to moderate degree (30%–50% knockdown in shTopBP1-1A and 1B cells) enhanced Chk1 activity after DNA replication stress, whereas an almost complete knockdown of TopBP1 (80%–100% knockdown in shTopBP1-2 and 3 cells) greatly eliminated Chk1 phosphorylation (Fig. 2*A*). We also performed a time course experiment by treating these stable cells with HU for 1, 2, or 5 h and obtained the same conclusion (Fig. 2*B*). These data suggest that, although TopBP1 is required for ATR/Chk1 activation in response to replicative stress, depletion of TopBP1 to a moderate level in cancer cells that express high levels of TopBP1, such as MDA-MB468, can actually induce a stronger ATR/Chk1 response to replicative stress.

To further support this notion, we reconstituted TopBP1 in one of the MDA-MB468 cell lines that showed modest knockdown of TopBP1 (shTopBP1-1A cells) by Ad-TopBP1 infection at various MOIs, such that their TopBP1 expression was lower or close to that in the shScr control cells. We found that modest (less than 50%) depletion of TopBP1 enhanced Chk1 phosphorylation induced by HU treatment for 5 h; nevertheless, reconstitution of TopBP1 to the level comparable with that in the control cells could reverse this effect (Fig. 2*C*). Taken together, these results indicate that an optimal level of TopBP1 is required for ATR/Chk1 activation. In cancer cells, the levels of TopBP1 affect the amplitude of Chk1 activation in response to DNA damage. When TopBP1 levels reach above an optimal level, it can in fact paradoxically decrease the degree of Chk1 activation.

### High levels of TopBP1 perturb its binding to ATR and decrease the levels of chromatin-associated ATR

**Figure 3:**
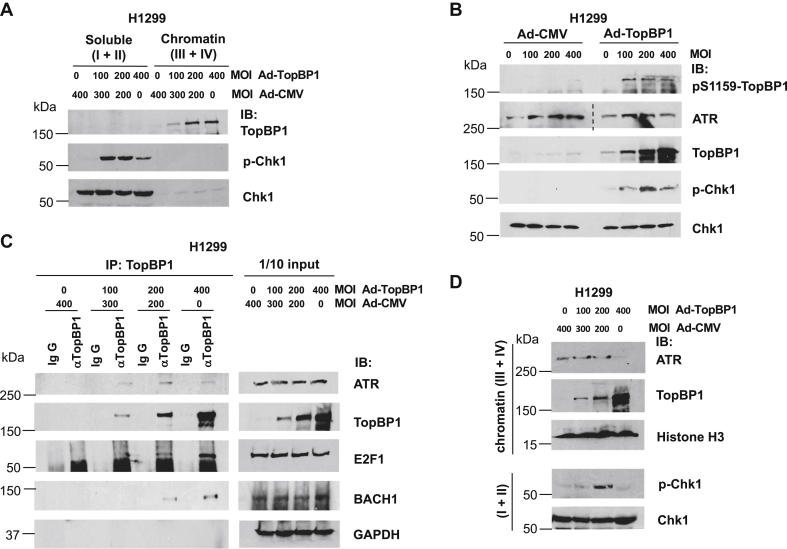
**Excessive TopBP1 can inhibit Chk1 activation induced by hydroxyurea (HU) by interfering with its binding to ATR**. *A*, H1299 cells stably expressing a TopBP1 shRNA was infected with Ad-TopBP1 at the indicated multiplicity of infection. Ad-CMV was used to equalize the total amount of virus for each sample. After 48 h, cells were treated with HU (2 mM) for 5 h, followed by chromatin fractionation. The combined soluble fractions (fractions I and II) and chromatin fractions (fractions III and IV) from each group were resolved by SDS-PAGE, and immunoblotting was performed using the indicated antibodies. *B*, TopBP1-depleted H1299 stable cells were infected with either Ad-CMV or Ad-TopBP1 at the indicated multiplicity of infection (MOI). Forty-eight hours later, cells were treated with HU (2 mM) for 5 h and then subjected to Western blotting. The same lysates were rerun for ATR immunoblotting, and a dashed line denotes the junction where a space was excised for presentation. *C*, H1299 cells stably expressing a TopBP1 shRNA were infected with Ad-TopBP1 and/or Ad-CMV as described in *A*. After HU treatment for 5 h, immunoprecipitation was performed using an anti-TopBP1 mouse monoclonal antibody or control mouse IgG, followed by immunoblotting to detect the associated ATR, E2F1, or BACH1. One-tenth of cell lysates were subjected to Western blot analysis. *D*, TopBP1-depleted H1299 cells were infected with Ad-TopBP1 and/or Ad-CMV as described in *A*. After HU treatment for 5 h, a chromatin binding assay was performed to examine ATR binding to chromatin.

To study the underlying molecular mechanism by which high levels of TopBP1 lead to declined Chk1 activity during DNA damage, we first tested whether highly expressed TopBP1 could interfere with its binding to chromatin, thereby inhibiting the TopBP1/ATR/Chk1 pathway. To test this possibility, we infected TopBP1-depleted H1299 cells with Ad-TopBP1 at various MOIs and then treated cells with HU, followed by chromatin fractionation (fractions III + IV representing chromatin fractions (19)). As shown in Figure 3*A*, the effect of TopBP1 on enhancing HU-induced Chk1 activation started to decline when the MOI of Ad-TopBP1 reached 400. Under this condition, TopBP1 could still tightly bind to chromatin. Since phosphorylation of TopBP1 at the S1159 residue by Akt can induce TopBP1 oligomerization and hamper its ability to activate ATR (10), we next examined if S1159 phosphorylation of TopBP1 was increased when the levels of p-Chk1 started to decline. However, we did not see an increase of pS1159-TopBP1 levels by Ad-TopBP1 infection at MOI 400 *versus* 200, despite a decrease of p-Chk1 in cells infected at MOI 400 (Fig. 3*B*). This result excludes the possibility of Akt regulation in this process. Increasing the levels of TopBP1 also did not significantly alter ATR expression.

**Figure 4:**
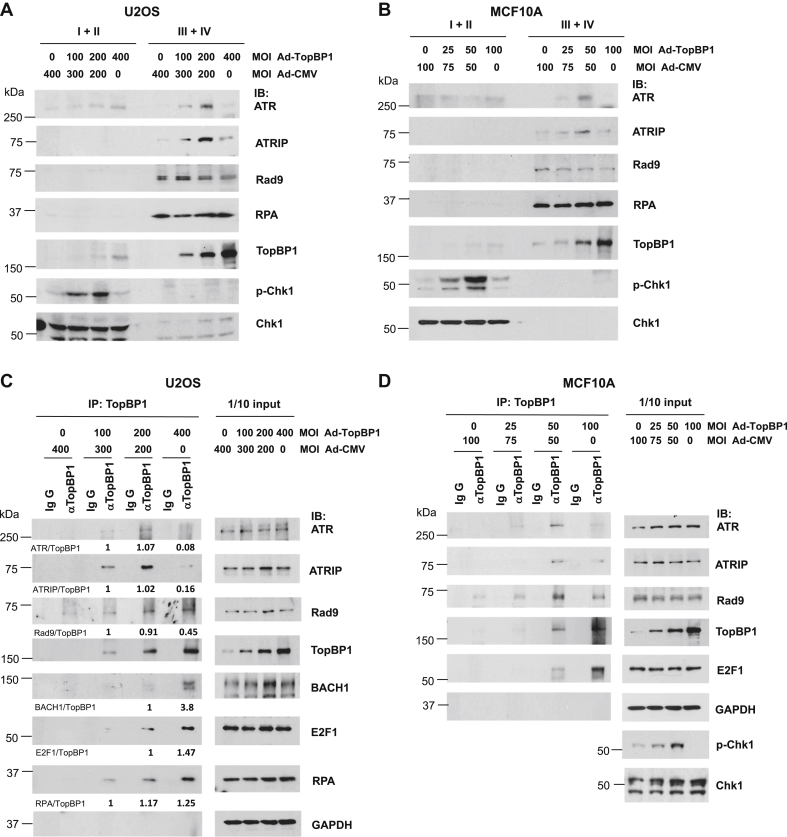
**TopBP1 overexpression inhibits chromatin recruitment of the ATR–ATRIP complex and the interaction of TopBP1 with ATR–ATRIP upon hydroxyurea treatment.** U2OS or MCF10A cells were infected with Ad-CMV or Ad-TopBP1 at the indicated MOI. Cells were then treated with HU (2 mM), followed by either chromatic fractionation as described in Figure 3*A* (*A* and *B*) or coimmunoprecipitation as described in Figure 3*C* (*C* and *D*). The relative intensities of immunoprecipitated proteins in (*C*) were quantified using ImageJ software and were normalized to the immunoprecipitated TopBP1 in the same samples.

Since the interaction between TopBP1 and ATR is required for TopBP1 to activate ATR, we next infected TopBP1-depleted H1299 cells with Ad-TopBP1 at various MOIs and then examined this binding following HU treatment for 5 h. As shown in Figure 3*C*, more ATR was coimmunoprecipitated with TopBP1 when TopBP1 levels were increased by Ad-TopBP1 infection from an MOI of 100 to 200, coinciding with the increased Chk1 phosphorylation. However, this interaction was decreased when the Ad-TopBP1 titer was increased to an MOI of 400 (Fig. 3*C*), coinciding with the declined Chk1 phosphorylation (Fig. 3*D*). In contrast, the association of TopBP1 with E2F1 or BACH1 was increased by elevating the titer of Ad-TopBP1 from an MOI of 100 to 400 (Fig. 3*C*). Moreover, chromatin binding assay demonstrated that ATR binding to chromatin was significantly decreased when TopBP1 was highly expressed by Ad-TopBP1 infection at an MOI of 400, concurrent with an increase of chromatin-associated TopBP1 and a declining level of Chk1 phosphorylation (Fig. 3*D*). These results could also be reproduced in two additional cell lines, U2OS and MCF10A, where expression of TopBP1 by Ad-TopBP1 caused biphasic changes of both Chk1 activation and ATR–ATRIP recruitment to chromatin (Fig. 4, *A* and *B*). In contrast, RPA recruitment to chromatin was not affected by TopBP1. On the other hand, Rad9 recruitment was slightly inhibited by TopBP1 in a dose-dependent, but not biphasic, manner. Consistently, coimmunoprecipitation showed that high levels of TopBP1 paradoxically inhibited its interaction with the ATR–ATRIP complex and to a lesser degree with Rad9, but not with BACH1, E2F1, or RPA (Fig. 4, *C* and *D*). Collectively, these data suggest that, when TopBP1 is expressed above an optimal level, it can paradoxically inhibit Chk1 activation after replicative stress through the inhibition of its interaction with ATR–ATRIP and interference of ATR–ATRIP recruitment to chromatin.

### BRCT1/2/3, but not S1159, phosphorylation of TopBP1 is required for the paradoxical inhibition of ATR by TopBP1 overexpression

**Figure 5:**
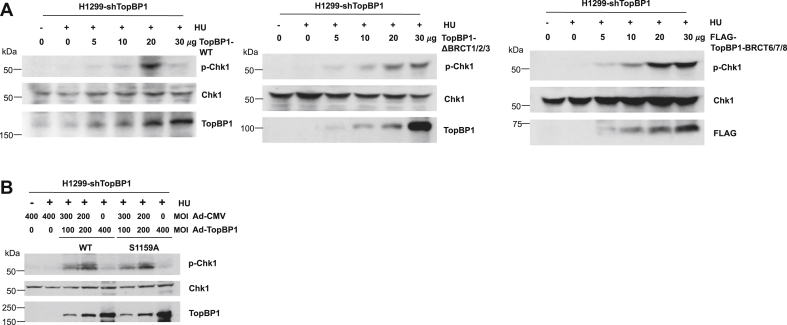
**The effect of TopBP1 overexpression on the inhibition of HU-induced Chk1 activation requires its BRCT1/2/3 domains but not Akt-mediated S1159 phosphorylation**. *A*, H1299-shTopBP1 cells were transfected with different amounts of plasmid expressing either WT TopBP1 or a deletion mutant of TopBP1 (ΔBRCT1/2/3 or FLAG-BRCT6/7/8). After HU treatment (2 mM) for 2 h, immunoblotting was performed to detect the expression of p-Chk1, Chk1, WT, or mutant TopBP1. *B*, H1299-shTopBP1 cells were infected with Ad-CMV and/or Ad-TopBP1 (WT or S1159A) at the indicated MOI. Cells were treated with HU (2 mM), followed by immunoblotting as described above. HU, hydroxyurea.

TopBP1 and ATR–ATRIP are recruited to stalled replication forks through binding to RPA-ssDNA (6). Too many TopBP1 molecules may saturate the available RPA-coated ssDNA on stalled replication forks and therefore prevent ATR–ATRIP recruitment. Since TopBP1 binds to RPA-ssDNA through its BRCT2 domain (6), we next investigated if overexpression of a TopBP1 mutant lacking BRCT2 fails to inhibit ATR/Chk1 activation. Indeed, in contrast to the wildtype TopBP1, a TopBP1-ΔBRCT1/2/3 mutant did not elicit the biphasic p-Chk1 effect after HU treatment (Fig. 5*A*). Previously we have shown that Akt-mediated phosphorylation of TopBP1 at S1159 induces TopBP1 oligomerization and inhibits its ability to activate ATR (10). However, a TopBP1-S1159A mutant that cannot be phosphorylated by Akt was capable of promoting the biphasic p-Chk1 response following HU treatment (Fig. 5*B*), indicating that Akt-induced oligomerization of TopBP1 is not involved in this process.

### A modest depletion of TopBP1 in cancer cells enhances S-phase checkpoint in response to HU treatment

**Figure 6:**
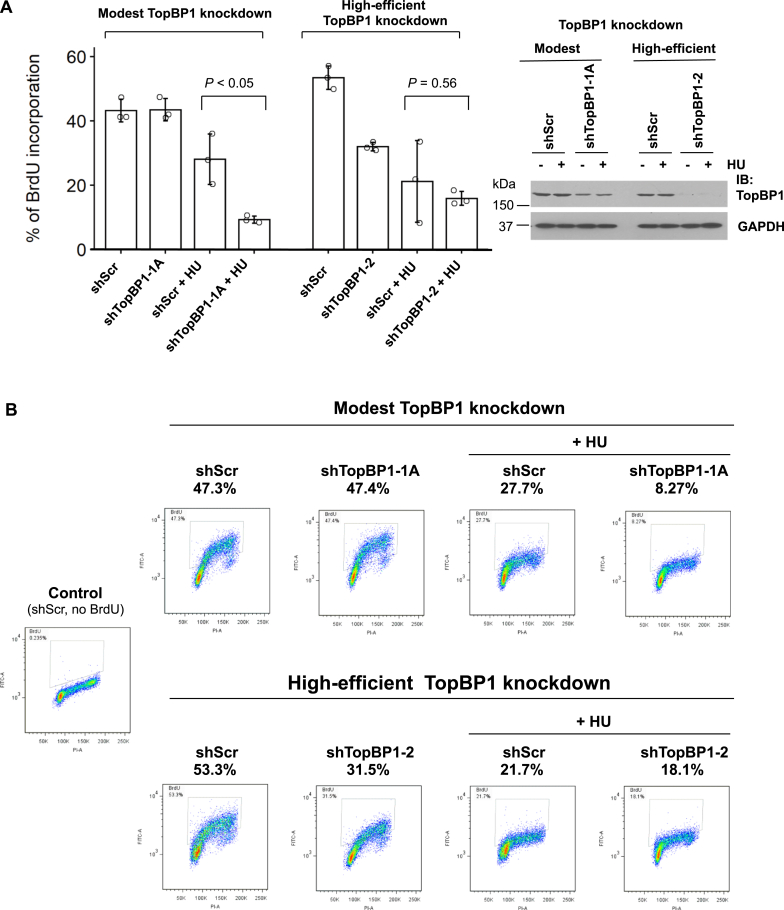
**Modest depletion of TopBP1 in the cells highly expressing TopBP1 enhances S-phase checkpoint response to HU treatment**. *A*, BrdU incorporation assay was performed in MDA-MB468 stable cells with modest or high-efficient knockdown of TopBP1. Cells were treated with HU (2 mM) or DMSO for 2 h, followed by labeling with BrdU (10 μM) for another 2 h. Cells were then collected and fixed for anti-BrdU antibody staining. Flow cytometric analysis was performed to quantify BrdU incorporation in at least 10,000 cells per sample. Data shown represent means ± SD from three biological replicates. The *p* values are based on a two-tailed *t* test. The effect of TopBP1 knockdown was verified by immunoblotting. *B*, representative profiles of BrdU incorporation described in (*A*). HU, hydroxyurea.

When cells are exposed to DNA replicative stress like HU, the cell cycle checkpoint will be activated and DNA synthesis will be halted to allow cells to repair the damaged DNA before it moves to the next phase of cell cycle. Based on the results shown above, we predicted that inhibition of HU-induced Chk1 activation by excessively expressed TopBP1 would lead to the impairment of checkpoint activation and cell cycle arrest. To investigate the physiological role of TopBP1 in the checkpoint response in cancer cells expressing high levels of TopBP1, we next performed BrdU incorporation assay to examine how a modest (less than 50%) or high (more than 90%) degree of TopBP1 depletion may affect DNA synthesis after HU treatment in TopBP1-depleted MDA-MB468 stable cells. The incorporated BrdU was both quantified by flow cytometry (Fig. 6, *A* and *B*) and visualized under fluorescence microscope (see Fig. S1). We found that modest depletion of TopBP1 did not affect BrdU incorporation in the absence of HU but potentiated the reduction of BrdU incorporation after HU treatment (Fig. 6, *A* and *B*), suggesting that reduction of TopBP1 may potentiate the S-phase checkpoint response in MDA-MB468 cells. In parallel, we also measured the response in high-efficient TopBP1 knockdown MDA-MB468 cells (Fig. 6*A*, *right panel*). As expected, the BrdU incorporation was inhibited by high-efficient TopBP1 depletion even in the absence of HU. However, there was no significant change in BrdU incorporation between shScr control cells and the high-efficient TopBP1 knockdown cells after HU treatment, consistent with a reduction of p-Chk1 activation in these shTopBP1 cells (Fig. 2*A*, shTopBP1-2 and shTopBP1-3). Thus, in cancer cells with high levels of endogenous TopBP1, modest but not high-efficient knockdown of TopBP1 can render cells more responsive to replicative stress to halt DNA synthesis and allow repair of DNA damage as in normal cells.

### TopBP1 overexpression renders cells more sensitive to replication stress

**Figure 7:**
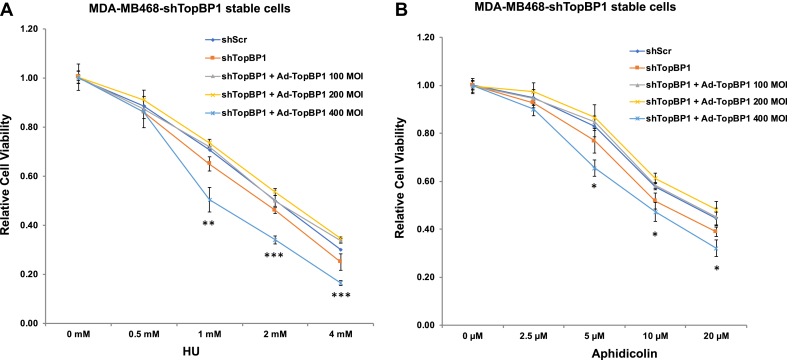
**TopBP1 overexpression makes cells more sensitive to replication stress inducers.** TopBP1-depleted MDA-MB468 cells were infected with Ad-CMV and/or Ad-TopBP1 at the indicated MOI as described in Figure 1*A*. On the next day, cells were plated onto 96-well plates. After settlement overnight, cells were treated with various concentrations of HU (*A*) or Aphidicolin (*B*) for 48 h, and cell viability was determined by MTT assay. Shown are means ± SD from three biological replicates. ∗*p* < 0.05; ∗∗*p* < 0.01; ∗∗∗*p* < 0.001, compared with corresponding shScr (two-tailed *t* test). HU, hydroxyurea; MOI, multiplicity of infection.

Inhibition of ATR sensitizes cells to replication stressors, such as HU and aphidicolin. To address the biphasic regulation of ATR activity by TopBP1, we next reconstituted TopBP1 at different levels in TopBP1-depleted MDA-MB468 cells and assessed cell viability after treatment with HU (Fig. 7*A*) or aphidicolin (Fig. 7*B*). Indeed, knockdown of TopBP1 increased cell sensitivity to HU or aphidicolin, and this effect could be rescued by adding Ad-TopBP1 back at MOI 100 or 200. However, when TopBP1 was reconstituted with Ad-TopBP1 at MOI 400, this rendered cells more sensitive to HU or aphidicolin, consistent with its effect on ATR inhibition.

### High levels of TopBP1 are associated with genome instability in cancer

**Figure 8:**
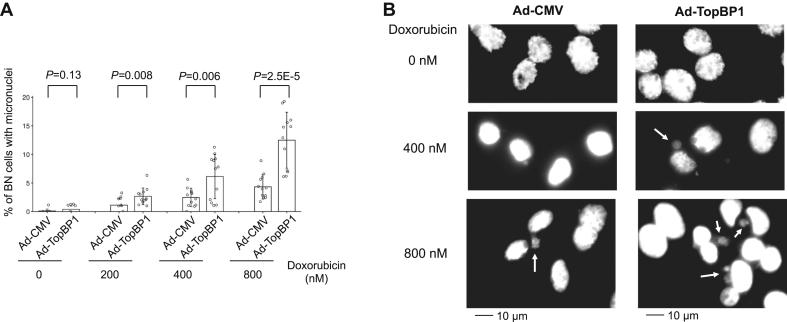
**Overexpression of TopBP1 impairs genome stability in MCF10A cells.** MCF10A cells were infected with Ad-CMV or Ad-TopBP1 at multiplicity of infection 100. The cytokinesis-block micronucleus assay was then performed as described in Experimental procedures to determine the frequency of binucleated (BN) cells with micronuclei after treatment with various doses of doxorubicin. After fixation, nuclei were stained with Hoechst 33258. *A*, images of four randomly selected fields with more than 100 binucleated cells per field were taken using a 20× objective under fluorescence microscope. The percentage of binucleated cells with micronuclei was quantified. Data represent means ± SD of 12 fields from triplicate samples with all raw values shown as open circles on the bar graph (*A*). *p* Values are calculated from a two-tailed *t* test. *B*, representative images were taken using a 40× objective. *Arrows* point to micronuclei. Scale bars are shown at the bottom of the figures.

We next examined whether TopBP1 overexpression affects genome integrity using the cytokinesis-block micronucleus assay (20, 21), which has been well established for the measurement of genomic instability. The data showed that overexpression of TopBP1 in MCF10A significantly increased the frequency of micronuclei-containing binucleated cells after a short treatment with doxorubicin (Fig. 8). This result provides evidence that overexpression of TopBP1 in normal cells can impair their ability to maintain genome stability, consistent with their effect on ATR/Chk1 inhibition.

**Figure 9:**
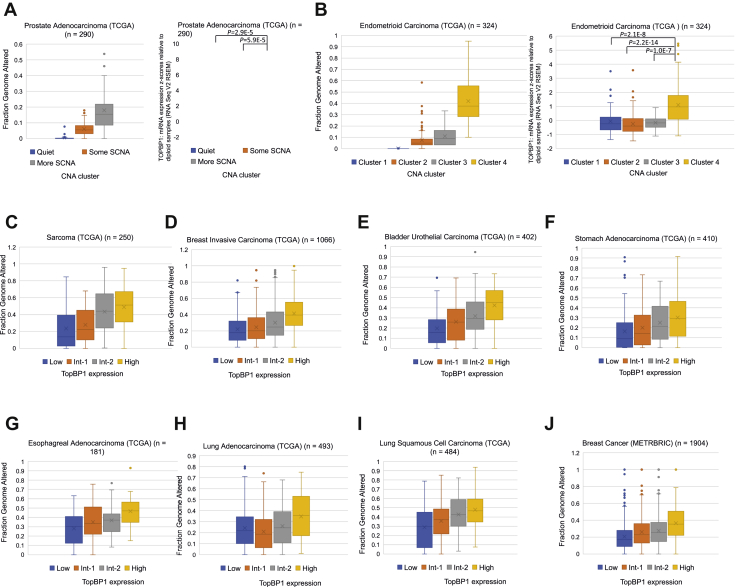
**High levels of TopBP1 are associated with genome instability in cancer**. *A*, *Left*: fraction of genome altered in three CNA clusters defined in prostate cancer TCGA study (22). *Right*: Expression of TopBP1 in each CNA cluster. *B*, *Left*: fraction of genome altered in four CNA clusters defined in TCGA endometrial carcinoma paper (23). *Right*: Expression of TopBP1 in each CNA cluster. *C–I*, tumor samples in each TCGA database were ranked according to the levels of TopBP1 mRNA and then were equally divided into four groups (Low, Int-1, Int-2, and High) for analysis of the fractions of genome altered. The one-way ANOVA *p* values for four groups in all graphs are <0.0001. The *p* values for the comparison between High and Low groups are also <0.0001 in all cancer types. *J*, similar analysis in (*C–I*) was performed using the METABRIC breast cancer dataset. The one-way ANOVA *p* value for four groups is <0.0001. The *p* value for the comparison between High and Low groups is also <0.0001. CAN, copy number alteration; SCNA, somatic copy number alteration; TCGA, The Cancer Genome Atlas Program.

To obtain further evidence for the association between high TopBP1 expression and genome instability in cancer, we explored several cancer genome databases. In both prostate and uterine endometrioid carcinoma The Cancer Genome Atlas Program (TCGA) cohorts (22, 23), the patients have been clustered based on the somatic copy number alterations (SCNAs). We analyzed TopBP1 expression in the copy number alterations (CNAs) subtypes that are already grouped in both studies (Fig. 9, *A* and *B*, *right panels*). As a positive control for the clustering, we also compiled the fraction of genome altered (percentage of genome affected by copy number gains or losses; cBioportal) in each sample in these clusters (Fig. 9, *A* and *B*, *left panels*). Indeed, high TopBP1 expression is associated with more SCNA in prostate cancer (Fig. 9*A*). In TCGA uterine corpus endometrioid carcinoma, high levels of TopBP1 are also significantly found in cluster 4, which is characterized by a very high degree of SCNAs (Fig. 9*B*) (23).

We next used the fraction of genome altered in each sample as an index for genomic instability to investigate its correlation with TopBP1 expression across all cancer types in PanCancer TCGA datasets. We found very good correlations between TopBP1 expression and Fraction Genome Altered in the majority of cancer types in PanCancer TCGA (Fig. 9, *C*–*I*) and METABRIC breast dataset (Fig. 9*J*), suggesting that this association is a general phenomenon among different cancer types.

## Discussion

A full function of ATR/Chk1 activation is of paramount importance for genome stability. Although a role for TopBP1 in ATR/Chk1 activation in normal cells is well established, the significance of TopBP1 overexpression in ATR/Chk1 activation in cancer cells expressing high levels of TopBP1 has not been investigated. Here we uncover an unexpected biphasic concentration-dependent response of ATR/Chk1 activation controlled by the levels of TopBP1 in cancer cells. By reconstitution of TopBP1 in the TopBP1-deleted cancer cells with different MOI of Ad-TopBP1, we demonstrate that low levels of TopBP1 stimulate, but high levels of TopBP1 inhibit, Chk1 activation. The mechanistic analysis further reveals that, when TopBP1 accumulates above an optimal level, it can paradoxically decrease the chromatin-associated ATR and its interaction with ATR, resulting in reduced ATR/Chk1 activation. The result is very relevant in cancer, since a modest reduction of TopBP1 level in cancer cells that highly express TopBP1 can enhance S-phase checkpoint response to replicative stress. Thus, many cancer cells may not have full functionality of ATR response owing to overexpression of TopBP1. Therapy that can reduce TopBP1 levels might refortify the checkpoint response in many cancers that overexpress TopBP1.

High TopBP1 expression may contribute to tumor development by reducing the strength of ATR/Chk1 activation. A complete deficiency of Chk1 is lethal, whereas conditional heterozygous knockout of *Chk1* in mouse mammary glands causes inappropriate S phase entry, accumulation of DNA damage, and premature mitosis, demonstrating Chk1 as a haploinsufficient tumor suppressor (24). Thus, the checkpoint function of Chk1 is dependent on its full expression. Similar to Chk1, ATR also acts as a haploinsufficient tumor suppressor (25). Hence, the attenuation of ATR/Chk1 activation by TopBP1 overexpression is expected to contribute to tumorigenesis through genomic instability. In fact, high levels of TopBP1 expression are strongly associated with genome instability in many types of cancer (Fig. 9).

Of interest, the biphasic pattern of interaction is only seen in the TopBP1–ATR complex but not in the complex of TopBP1 with E2F1, BACH1, or RPA (Figs. 3*C* and 4, *C* and *D*). As more TopBP1 is overexpressed, there are more TopBP1–E2F1 and TopBP1–BACH1 complexes. This result demonstrates that the interaction of TopBP1 with ATR is distinct from its binding to either E2F1 or BACH and is more dependent on appropriate and modest levels of TopBP1. As TopBP1 expression is increased, the chromatin-bound TopBP1 is also increased (Figs. 3*A* and 4, *A* and *B*). Since both TopBP1 and ATR–ATRIP are recruited to stalled replication forks through binding to RPA-coated ssDNA (6), when the chromatin-bound TopBP1 accumulates above an optimal level, too many TopBP1 molecules may saturate the available RPA-coated ssDNA on a stalled replication fork and therefore prevent ATR–ATRIP recruitment. This possibility is supported by the data that TopBP1-ΔBRCT1/2/3 does not exhibit the biphasic response (Fig. 5*A*). In addition, binding to both 9-1-1 and ATR–ATRIP is required for TopBP1 to activate ATR (8). Excessive TopBP1 on stalled replication forks may prevent TopBP1 from forming proper complexes with both 9-1-1 and ATR–ATRIP (Fig. 4, *C* and *D*) simultaneously and presenting its ATR-activating domain for ATR activation. Future investigation will be needed to elucidate the structural basis of these interactions.

## Experimental Procedures

### Cell culture

MDA-MB468, H1299, C33A, and K562 cells were maintained in Dulbecco's modified Eagle's medium supplemented with 10% fetal bovine serum. OVCAR-3 cells were maintained in RPMI-1640 medium supplemented with 10% fetal bovine serum. MCF10A were maintained in Dulbecco's modified Eagle's medium/F12 supplemented with horse serum (5%), epidermal growth factor (20 ng/ml), hydrocortisone (0.5 μg/ml), cholera toxin (100 ng/ml), and insulin (10 μg/ml). Penicillin (50 IU/ml) and streptomycin (50 μg/ml) were added to all culture media. All cells were grown in a humidified incubator at 37 °C with 5% CO_2_ and 95% air.

### Establishment of stable cell lines

MDA-MB468 and H1299 cells were infected with lentivirus harboring a scrambled shRNA or a TopBP1 shRNA (16) followed by selection with puromycin (2 μg/ml) to establish stable cell lines expressing a scrambled shRNA (shScr) or a TopBP1 shRNA (shTopBP1). The effect of knockdown was confirmed by Western blotting using an antibody specific to TopBP1.

### Immunoprecipitation and Western blot analysis

Immunoprecipitation was performed by incubating equal amounts of cell lysates with an anti-TopBP1 mouse monoclonal antibody or control mouse IgG and then with protein A/G agarose beads (from GenDepot) for 16 h at 4 °C. After three washes, immunoprecipitates were fractionated by SDS-PAGE and electrotransferred to Imobilon-P membrane (Millipore). Immunoblotting was performed with appropriate antibodies. Antibody specific to E2F1 (C-20 or KH-95), Chk-1 (G-4), Rad9 (M-389), or GAPDH (6C5) was from Santa Cruz Biotechnology. Anti-TopBP1 mouse monoclonal antibody was from BD Transduction Laboratories. Anti-TopBP1 (BL893) rabbit polyclonal antibody was from Bethyl Laboratories. Anti-phospho-TopBP1 (S1159) antibody was from Abgent. Antibody specific to p-Chk1(S345), ATR, ATRIP, BACH1/BRIP1, RPA32, or Histone H3 was from Cell Signaling.

### Chromatin binding assay

The assay was performed as described (10). Briefly, the infected cells were first resuspended for 5 min on ice in 150 μl fractionation buffer (50 mM Hepes, pH 7.5, 150 mM NaCl, 1 mM EDTA) containing 0.2% NP-40, supplemented with protease inhibitors as described. Following centrifugation at 1000*g* for 5 min, the supernatant was collected (fraction I), and pellets were washed with the same buffer. The wash was collected (fraction II), and the nuclear pellets were further extracted for 40 min on ice with 150 μl fractionation buffer containing 0.5% NP-40. The extracts were clarified by centrifugation at 16,000*g* for 15 min (fraction III). The pellets were finally lysed in 150 μl of 10% SDS-PAGE sample buffer and boiled for 5 min (fraction IV). The aliquots of each fraction derived from equivalent cell numbers were collected. In this study, the combined aliquots from fraction (I+II) and fraction (III+IV) were separated on 10% SDS-PAGE, and Western blotting was carried out.

### Bromodeoxyuridine incorporation assay and flow cytometry

For flow cytometric analysis, cells were labeled with 5-bromo-2-deoxyuridine (BrdU, 10 μM) for 2 h and then fixed with 70% ethanol. Cells were treated with 2 N HCl/Triton X-100 for 30 min and then neutralized with 0.1 M sodium tetraborate, pH 8.5. Cells were then stained with FITC-conjugated anti-BrdU antibody (BD Biosciences) and propidium iodide, followed by flow cytometry. At least 10,000 cells were analyzed for each sample. All experiments were performed in at least triplicates. For microscopic analysis, cells were labeled with BrdU for 6 h. After fixation with 4% formaldehyde, the incorporated BrdU was detected with an anti-BrdU antibody (Ab-3, Calbiochem) followed by Texas Red X-conjugated secondary antibody (Invitrogen). Nuclei were stained with Hoechst 33258 dye. Images were captured with a Zeiss fluorescence microscope (Axio Observer Inverted Microscope).

### Statistical analysis

Two-tailed *t* test was performed to compare two experimental groups. One-way ANOVA was used to determine whether three or four groups are statistically different from each other. Data were presented as means ± SD from at least three biological replicates. *p* Values less than 0.05 were considered statistically significant. RNA sequencing (RNA-Seq) gene expression data, CNA clustering, and fraction of genome altered in TCGA database were extracted from cBioportal server (https://cbioportal.org/). Gene expression and CNA data in the METABRIC breast cancer dataset were also extracted from cBioportal server. The fraction of genome altered in each sample was then calculated from the METABRIC CNA dataset based on the fraction of 22,544 genes/genomic loci with copy number gains or losses. Pearson correlation coefficients were calculated to evaluate correlations.

### MTT assay

MTT assay was used to determine cell viability. Cells were seeded in 96-well plates at 5000 to 10,000 cells per well for 24 h, followed by indicated treatment for another 48 h. Cells were then washed and incubated with MTT solution (Thiazolyl blue tetrazolium bromide, 5 mg/ml) (Sigma) at 37 °C for 3 to 4 h. After the removal of medium and MTT solution, 100 μl DMSO was added to each well. The absorbance was read at 570 nm on a plate reader (BioTek Synergy HT). Each experiment was performed at least in triplicates.

### Cytokinesis-block micronucleus assay

MCF10A were infected with Ad-CMV or Ad-TopBP1 at MOI 100. Twenty-four hours later, cells were split into six-well plates. On the next day, cells were treated with doxorubicin at increasing doses for 4 h. Media were then removed and fresh medium containing cytochalasin B (3 μg/ml) was added for another 24 h. Cells were then fixed with 4% formaldehyde, and nuclei were stained with Hoechst 33258 dye. Images were captured with a Zeiss fluorescence microscope (Axio Observer inverted microscope). At least 1000 binucleated cells per group were scored. The frequency of micronuclei-containing binucleated cells among all binucleated cells was calculated.

## Data availability

All data are presented in the article.

## Supporting information

This article contains supporting information.

## Conflict of interest

The authors declare that they have no conflicts of interest with the contents of this article.
