# Supplementary material for: Overexpression of TopBP1, a canonical ATR/Chk1 activator, paradoxically hinders ATR/Chk1 activation in cancer
Source: J Biol Chem. 2021 Feb 5;296:100382. doi: 10.1016/j.jbc.2021.100382 (PMC7949146; doi:10.1016/j.jbc.2021.100382)

Supporting Information

Overexpression of TopBP1, a canonical ATR/Chk1 activator, paradoxically hinders ATR/Chk1 activation in cancer

Kang Liu, Joshua D. Graves, Fang-Tsyr Lin and Weei-Chin Lin

Content:

Supporting Information Fig. S1.

Fig. S1. Modest depletion of TopBP1 in the cells highly expressing TopBP1 enhances S-phase checkpoint response to HU treatment.

The BrdU incorporation assay was carried out as described in Fig. 6, except that MDA-MB468 cells were labeled with BrdU for 6 h and the incorporated BrdU was visualized and quantified under fluorescence microscope. Shown are means±SD from six biological replicates. *P* values are calculated from a two-tailed *t* test. Representative images are shown in Fig. S1B and Fig. S1C.

Fig. S1A

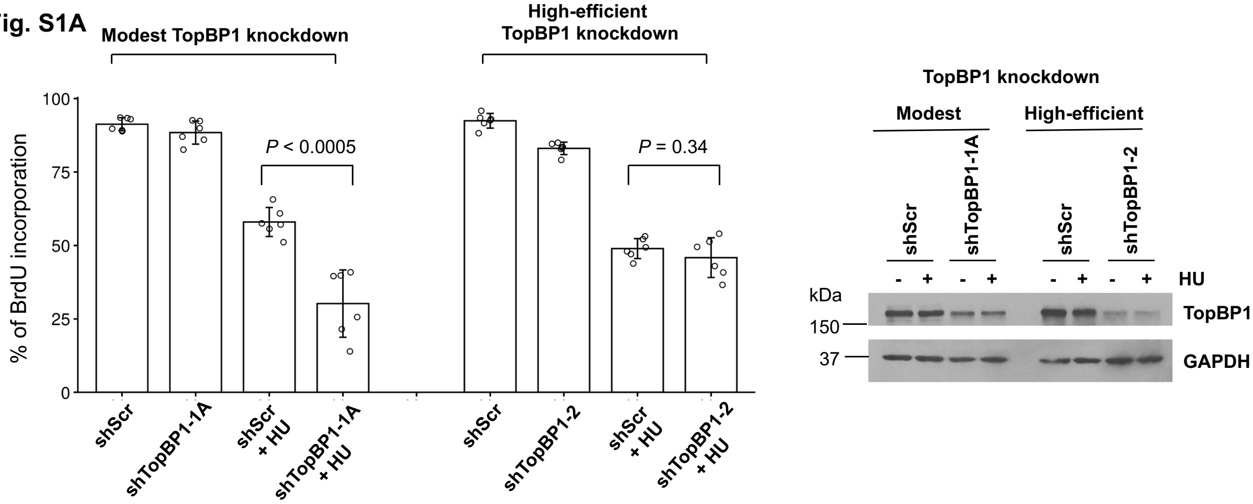

Fig. S1B

Modest TopBP1 knockdown

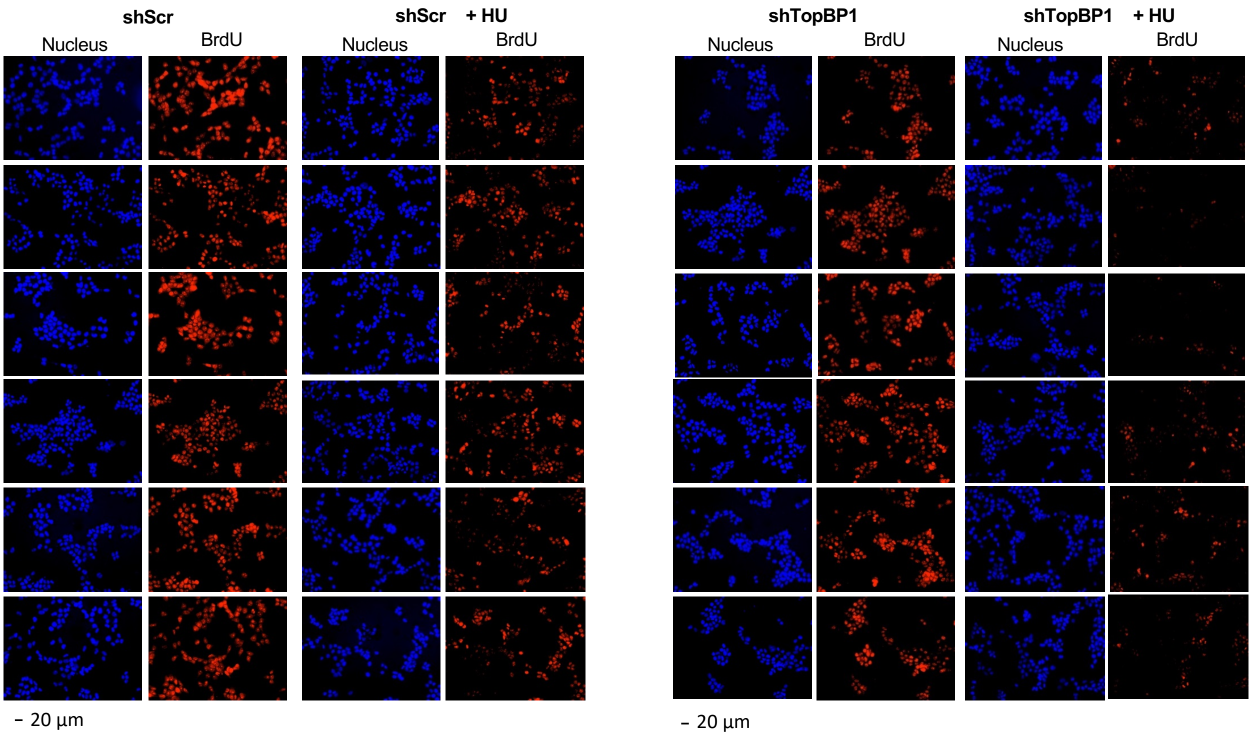

Fig. S1C

High-efficient TopBP1 knockdown

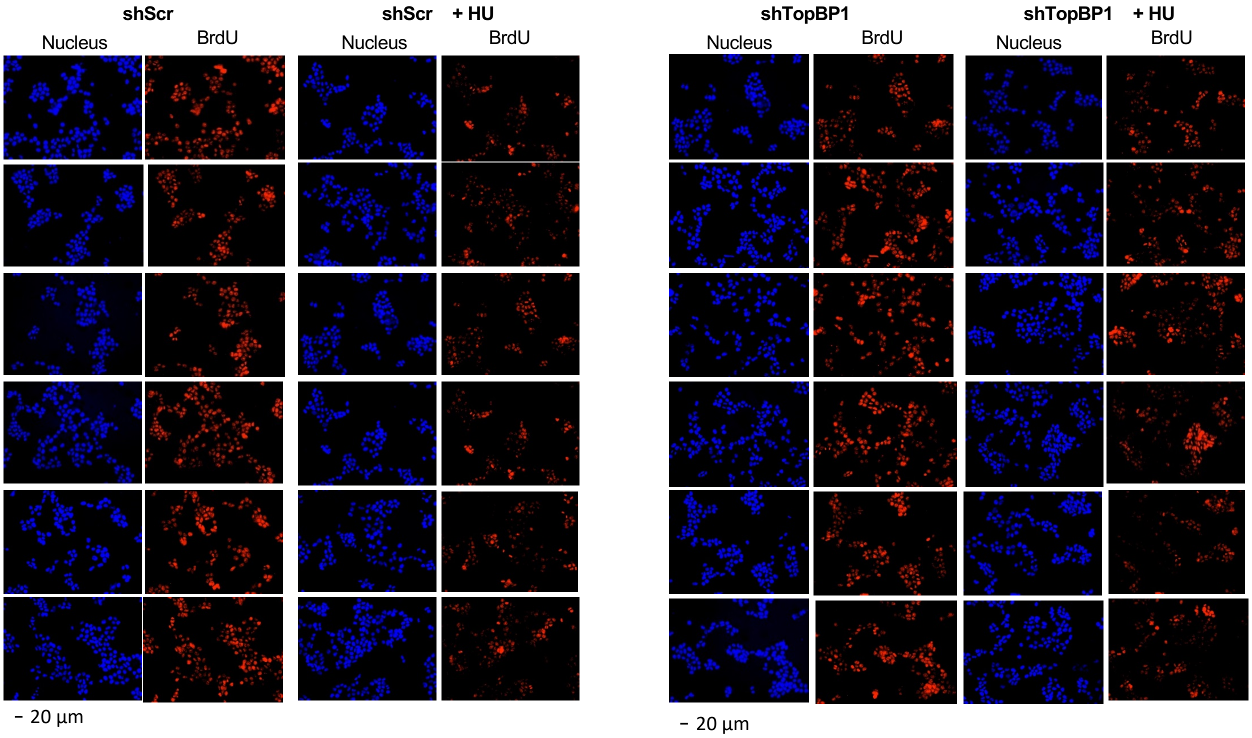

Supplement: Figure S1 [file mmc1.pdf]
